# Carnosol Modulates Th17 Cell Differentiation and Microglial Switch in Experimental Autoimmune Encephalomyelitis

**DOI:** 10.3389/fimmu.2018.01807

**Published:** 2018-08-13

**Authors:** Xing Li, Li Zhao, Juan-Juan Han, Fei Zhang, Shuai Liu, Lin Zhu, Zhe-Zhi Wang, Guang-Xian Zhang, Yuan Zhang

**Affiliations:** ^1^National Engineering Laboratory for Resource Development of Endangered Crude Drugs in Northwest China, Key Laboratory of the Ministry of Education for Medicinal Resources and Natural Pharmaceutical Chemistry, College of Life Sciences, Shaanxi Normal University, Xi’an, China; ^2^Department of Neurology, Thomas Jefferson University, Philadelphia, PA, United States; ^3^Department of Pharmacy, Zhengzhou University, Zhengzhou, China

**Keywords:** Carnosol, multiple sclerosis, experimental autoimmune encephalomyelitis, Th17 cell, macrophage/microglia

## Abstract

Medicinal plants as a rich pool for developing novel small molecule therapeutic medicine have been used for thousands of years. Carnosol as a bioactive diterpene compound originated from *Rosmarinus officinalis* (Rosemary) and *Salvia officinalis*, herbs extensively applied in traditional medicine for the treatment of multiple autoimmune diseases (1). In this study, we investigated the therapeutic effects and molecule mechanism of carnosol in experimental autoimmune encephalomyelitis (EAE), an animal model of multiple sclerosis (MS). Carnosol treatment significantly alleviated clinical development in the myelin oligodendrocyte glycoprotein (MOG_35–55_) peptide-induced EAE model, markedly decreased inflammatory cell infiltration into the central nervous system and reduced demyelination. Further, carnosol inhibited Th17 cell differentiation and signal transducer and activator of transcription 3 phosphorylation, and blocked transcription factor NF-κB nuclear translocation. In the passive-EAE model, carnosol treatment also significantly prevented Th17 cell pathogenicity. Moreover, carnosol exerted its therapeutic effects in the chronic stage of EAE, and, remarkably, switched the phenotypes of infiltrated macrophage/microglia. Taken together, our results show that carnosol has enormous potential for development as a therapeutic agent for autoimmune diseases such as MS.

## Introduction

Multiple sclerosis (MS) and its animal model, experimental autoimmune encephalomyelitis (EAE), are chronic immune-mediated demyelinating diseases of the central nervous system (CNS), characterized by infiltrated inflammatory cells, demyelination, and damage to neurons (2). Although the underlying mechanism of MS has not been well defined, a growing body of evidence supports its being an autoimmune disease (3). While Th1 cells have been considered pathogenic for MS/EAE, Th17 cells, a subpopulation of pro-inflammatory T helper cells defined by their secretion of IL-17 (4), have recently emerged as an important player in inflammatory and autoimmune diseases *via* the secretion of pro-inflammatory cytokines, such as IL-17A, IL-17F, GM-CSF, and IL-22 (5, 6). Polarization of Th17 populations and the related cytokine production are directly regulated by RORγt (7), and the signals that cause Th17 cells to differentiate actually inhibit regulatory T cell (Treg) differentiation (8). Therefore, targeted inhibition of RORγt transcription or a Th17 differentiation-related signaling pathway such as NF-κB and signal transducer and activator of transcription 3 (STAT3) represents an encouraging therapeutic strategy in treatment of Th17-related diseases (4, 9, 10).

Current MS therapies either have limited efficacy or important safety issues (11, 12). A great deal of research effort has gone into developing novel therapies that specifically target Th17 cells, while sparing other immune cells. Recently, several new anti-inflammatory or immunomodulatory drugs derived from medicinal plants have been explored and are considered to have great potential for treatment of autoimmune diseases (4, 13–15). These natural compounds represent a rich source for the identification of effective and safe candidate medicines with innovative targets and/or mechanisms of action in the therapy of MS and other autoimmune diseases.

*Rosmarinus officinalis* (rosemary) and *Salvia officinalis* are common household plants that grow all over the world and have been used as medicinal herbs due to their powerful antioxidant and anti-inflammatory effects (16, 17). Carnosol, a major diterpene present in *R. officinalis* (rosemary) and *S. officinalis*, has been reported to possess strong antioxidant, anti-tumor, anti-viral, and especially anti-inflammatory properties (18–20). Carnosol treatment also induced T-cell leukemia/lymphoma apoptosis and decreased IL-6 and TNF-α levels in serum (21, 22). These studies indicate that carnosol may be effective in the treatment of autoimmune diseases; however, this possibility has not been tested. To elucidate this question, in the present work, we studied the potential therapeutic anti-inflammatory abilities of carnosol on actively induced and adoptively transferred EAE models and the mechanism of its action.

## Materials and Methods

### EAE Induction and Treatment

Female C57BL/6 mice (purchased from the Fourth Military University (Xi’an, China)) were used at the age of 8 weeks. All animal experiments were performed with the approval of the Institutional Animal Care and Use Committee of Shaanxi Normal University and according to the approved institutional guidelines and regulations. For acute and chronic EAE, a previously described method was followed (23). Briefly, mice were subcutaneously injected with 200 µg of myelin oligodendrocyte glycoprotein (MOG) peptide 35–55 (Genescript, Piscataway, NJ, USA) in 200 µl of emulsified complete Freund’s adjuvant with 5 mg/ml *Mycobacterium tuberculosis* H37Ra (Difco, Lawrence, KS, USA). For adoptive transfer EAE, mice were sacrificed 10 days after MOG_35–55_ immunization, and splenocytes and draining lymph nodes were provided as previously described (4). Cells were cultured for 3 days in the presence of 25 µg/ml MOG_35–55_, 10 ng/ml rmIL-23, and 2 ng/ml rmIL-2 (R&D Systems, Minneapolis, MN, USA) at 1 × 10^7^ cell/ml. CD4^+^ T cells were purified by CD4^+^ T cell isolation kit and 4 × 10^6^ cells per mouse were transferred *via* intravenous (i.v.) injection. Pertussis toxin (200 ng/mouse) was injected intraperitoneally (i.p.) on days 0 and 2. Clinical EAE was assessed by daily scoring using a 0–5 scale as described previously (24). Carnosol was obtained from Sigma-Aldrich (St. Louis, MO, USA) and was injected (50 mg/kg/day) i.p. daily starting at day 0 p.i.

### Histological and Immunofluorescence Staining

Mice were euthanized at different time points after drug administration, and transcardially perfused with PBS. Tissues (brains and spinal cords) were collected for pathological assessment. Spinal cords were fixed with 4% paraformaldehyde overnight, cut into 5 µm sections and stained with H&E (hematoxylin and eosin) for inflammation and Luxol fast blue (LFB) for demyelination. Slides were examined and assessed following a previously described method (23).

For immunofluorescence, brain and spinal cord were cryopreserved in OCT compound (Tissue-Tek, Sakura Finetek, Japan) for frozen sections and cut into 12 µm sections (25). Immunofluorescence staining was performed using general methods and the appropriate dilutions of primary antibodies were applied. Immunofluorescence controls were routinely performed with incubations in which primary antibodies were omitted. Images were acquired by Nikon Eclipse E600 fluorescent microscopy (Nikon, Melville, NY, USA). For quantification of CD45^+^, MOG^+^, MBP^+^, iNOS^+^, Arg1^+^, and CD68^+^, 10 areas of the sections were selected and analyzed as previously described (23).

### Cytokine Measurement by ELISA

Splenocytes from EAE mice were prepared and cultured in triplicates in RPMI 1640 supplemented with 10% fetal bovine serum (Thermo Fisher Scientific) and stimulated with 25 µg/ml MOG_35–55_ for 3 days. Cell-free supernatants were harvested and analyzed for IFN-γ, IL-17, GM-CSF, IL-5, and IL-10 by ELISA Kits (R&D Systems).

### Mononuclear Cell (MNC) Preparation

Splenocytes of EAE mice were mechanically pushing spleen tissue through a 70 µm strainer (Falcon, Tewksbury, MA, USA) and treated with red blood cell (RBC) lysis buffer (Biolegend, San Diego, CA, USA) for 60 s. Collected cells were flushed with pre-cold PBS before stimulation. To collect MNC from CNS tissue, brain and spinal cords were administered with Liberase (Roche, Nutley, NJ, USA) for half hour and dissociated through a 70 µm strainer and flushed with pre-cold PBS. Cells were then separated by 70/30% percoll (Sigma-Aldrich) gradient method following previously described (26).

### *In Vitro* T Cell Polarization

Polarization of Th1, Th17, and Treg cells was induced *in vitro* following a previously described method (4). Naive 8-week-old female C57BL/6 mice were sacrificed and spleen tissue was dissociated to single cell. Mouse CD4 microbeads (Miltenyi Biotech Inc.) were used to purify the CD4^+^ T cells. Then, cells were cultured for 3 days under their respective polarizing conditions (27). Cells were stimulated for 3 days and examined on FACSAria (BD Biosciences).

### Flow Cytometry Analysis

For cell surface staining, fluorochrome-conjugated Abs to CD4 (BD Biosciences, San Jose, CA, USA) or isotype control Abs were added to cells for 30 min. For all intracellular staining, CNS-infiltrating MNCs or splenocytes were stimulated for 5 h with phorbol 12-myristate 13 acetate (50 ng/ml), ionomycin (500 ng/ml) (Sigma-Aldrich), and GolgiPlug (BD Biosciences). The staining procedure was performed following a previously described protocol (4). Data were analyzed with FlowJo software (Treestar, Ashland, OR, USA).

### Quantitative PCR

Total RNA from T cells or microglia cells was extracted by RNeasy Plus Mini Kit (QIAGEN, Valencia, CA, USA). cDNA was synthesized with QuantiTect Reverse Transcription Kit (QIAGEN). Quantitative PCR was performed in ABI Prism 7500 Sequence Detection System (Applied Biosystems, Foster City, CA, USA) using QuantiFast SYBR Green PCR Kit (QIAGEN). All experiments involving mRNA levels were normalized to glyceraldehyde 3-phosphate dehydrogenase and primers that were based on published cDNA sequences are listed in Table S2 in Supplementary Material.

### Western Blot

T cells were activated on 24-well plate under Th17 differentiation condition w/o carnosol 10 µg/ml for 18 h and were then collected. Cells were lysed by cell lysis buffer (Cell Signaling Technology, Danvers, MA, USA) supplemented with 1 mM phenylmethylsulfonyl fluoride (Cell Signaling Technology). All samples containing 15 µg total proteins were separated by 10% SDS-PAGE and transferred to polyvinylidene difluoride membrane (Pierce Chemical, Rockford, IL, USA). Membranes were blocked with 5% (w/v) nonfat dry milk powder in Tris-buffered saline (TBS) for 2 h at room temperature. This was followed by incubation at 4°C overnight with primary antibodies. Afterward, the membrane was washed three times in TBS plus Tween and incubated with the corresponding secondary antibodies (Cell Signaling Technology). The protein band was detected using Pierce ECL Western Blotting Substrate (Thermo Fisher Scientific, Waltham, MA, USA).

### Statistical Analysis

Data were analyzed using GraphPad Prism 6 software (GraphPad, La Jolla, CA, USA), and are presented as the mean ± SD. Significant differences in comparing multiple groups, data were analyzed by Tukey’s multiple comparisons test. All other statistical comparisons were done using nonparametric statistical tests. Differences with *p* values of less than 0.05 were considered significant.

## Results

### Carnosol Treatment Remarkably Alleviated Acute Clinical EAE

**Figure 1:**
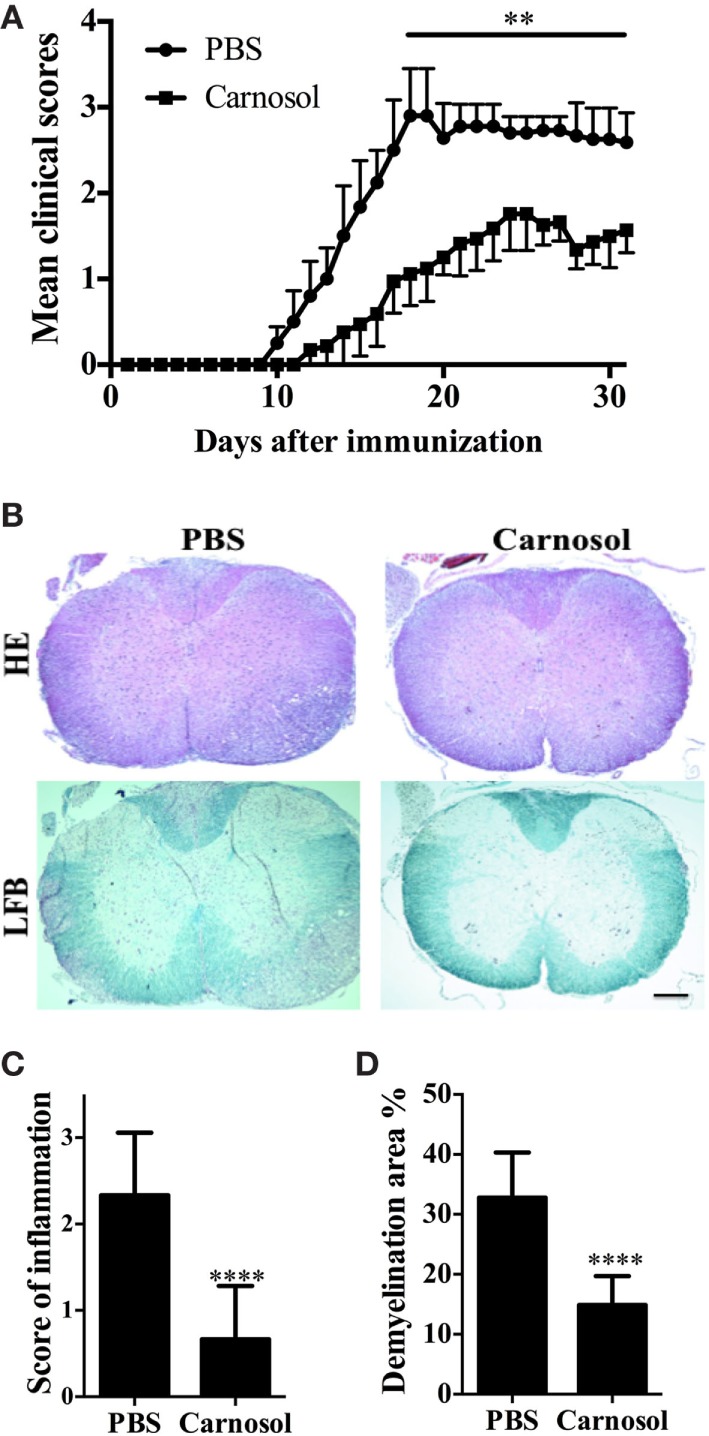
Carnosol ameliorated clinical severity of experimental autoimmune encephalomyelitis (EAE). C57BL/6 mice were injected i.p. with PBS or carnosol (50 mg/kg) daily starting on the day of EAE induction, and scored daily following a 0–5 scale **(A)**. **(B)** Mice were sacrificed at day 30 p.i. and spinal cords were harvested. Sections at lumbar level (L3) were analyzed by H&E and Luxol fast blue (LFB) (scale bar = 1 mm), and pathology scores of inflammation **(C)** and percentage of demyelination area **(D)** were evaluated. Data are mean ± SD (*n* = 5 each group). **p < 0.01 and ****p < 0.0001, determined by two-way ANOVA **(A)**, or nonparametric test **(C,D)**. One representative of three independent experiments is shown.

We first tested whether carnosol was effective in ameliorating the clinical severity of MOG-induced EAE by scoring disease signs daily on a 0–5 scale. The PBS-treated group of mice showed the first signs of EAE on day 10 p.i., while the carnosol-treated mice did so on day 12 p.i. Further, daily carnosol administration apparently led to decreased disease severity compared to the PBS-treated control group (*p* < 0.01; Figure 1A).

We then evaluated pathological changes by histologic analyses in lumbar spinal cords to examine CNS inflammatory infiltration and demyelination at day 30 p.i. As shown in Figure 1B, massive inflammatory infiltration and demyelination was observed in the spinal cord of PBS-treated EAE mice; by contrast, the carnosol-treated group displayed mild to moderate signs (*p* < 0.0001; Figures 1B–D). These results indicated that carnosol had a significantly suppressive effect in acute EAE.

### Carnosol Suppressed CNS Inflammation and Modulated Peripheral Immune Response in Acute EAE

**Figure 2:**
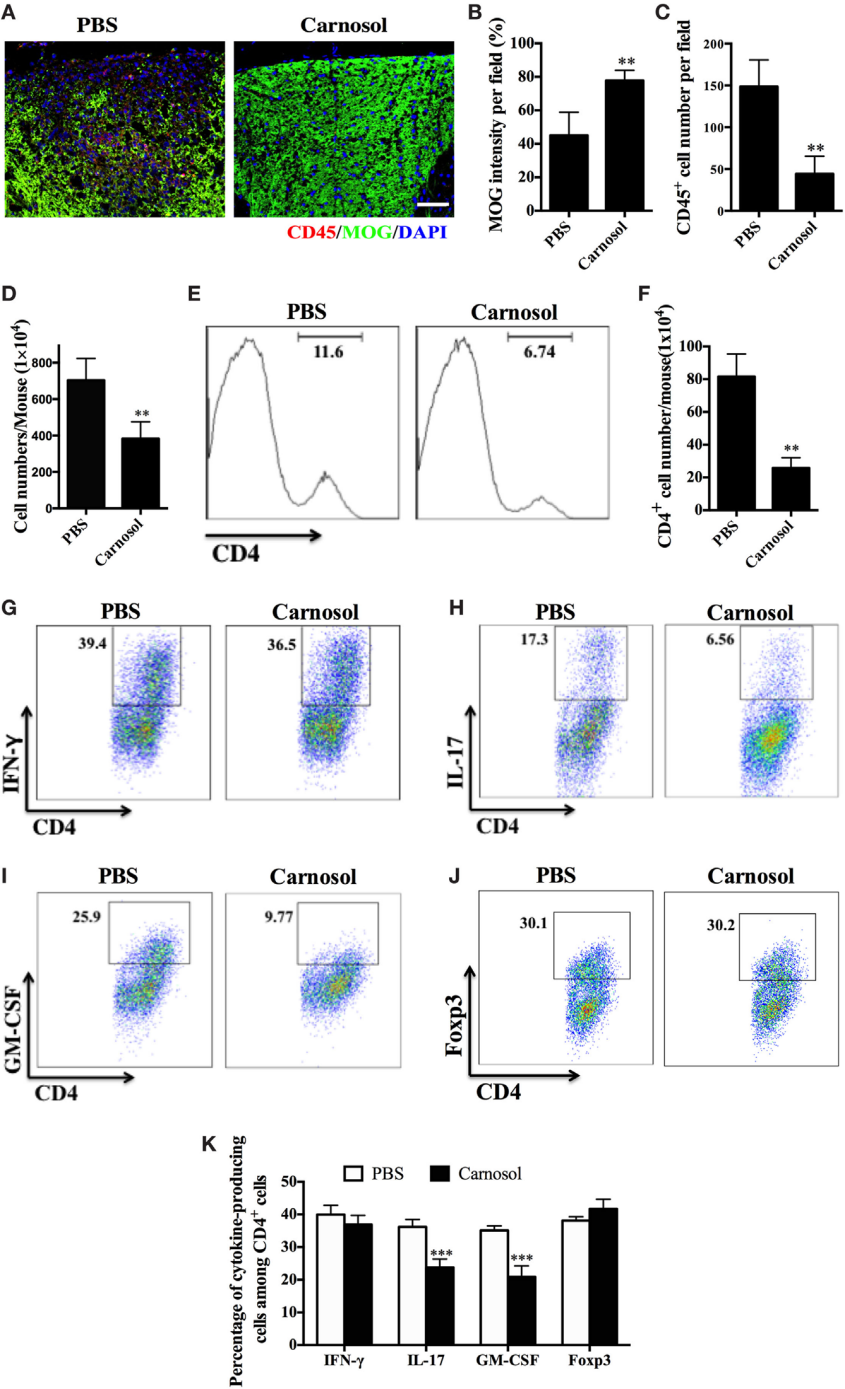
Carnosol treatment suppressed inflammatory infiltration in the central nervous system (CNS). Mice were treated with PBS or carnosol at the day of experimental autoimmune encephalomyelitis induction and sacrificed at day 30 p.i. **(A)** Spinal cords were subjected to immunostaining analysis. **(A)** Representative sections of thoracic spinal cord from PBS- and carnosol-treated mice were stained with CD45 and MOG (scale bar = 100 μm), and the number of CD45^+^ cells **(B)** and the intensity of MOG staining **(C)** were statistically analyzed. **(D)** Spinal cords and brains were harvested and mononuclear cells (MNCs) isolated (*n* = 10 each group). Total MNC numbers in CNS were counted under light microscopy. **(E)** The percentage of CD4^+^ T cells was measured by flow cytometry. **(F)** Absolute numbers of infiltrated CD4^+^ T were calculated by multiplying the percentages of these cells with total numbers of MNCs in each spinal cord and brain tissue. **(G–J)** Frequencies of IFN-γ^+^, IL-17^+^, GM-CSF^+^, and Foxp3^+^ cells among CD4^+^ cells were assessed by flow cytometry, and **(K)** the percentages of these cells in total CD4^+^ cell numbers in each CNS are shown. Symbols represent mean ± SD (*n* = 5 each group). **p < 0.01 and ***p < 0.001. Student’s *t*-test. One representative of three independent experiments is shown.

To evaluate the therapeutic effects of carnosol on CNS pathology, spinal cords were obtained from carnosol- and PBS-treated EAE mice. Analysis of spinal cord tissue sections showed abundant CD45^+^ inflammatory cells in the lesion area in the PBS-treated group, while these cells could barely be detected in the spinal cord tissue sections of carnosol-treated mice (*p* < 0.01; Figures 2A,B). Correspondingly, there was significantly reduced demyelination (MOG^−^ area) in carnosol-treated mice compared with the PBS-treated group (*p* < 0.01; Figures 2A,C). These results were consistent with the HE and LFB staining, indicating that carnosol inhibited inflammatory cell infiltration and demyelination in the CNS.

To further evaluate the effects of carnosol on the infiltrated inflammatory T cells into the CNS, MNCs were separated from the CNS and analyzed by flow cytometry. The total number of MNCs was 703.8 ± 119.0 × 10^4^ per mouse in the PBS-treated group vs. 382.6 ± 93.59 × 10^4^ in the carnosol-treated group (*p* < 0.01; Figure 2D). In addition, carnosol treatment significantly decreased the percentage and absolute numbers of CD4^+^ cells in the CNS compared to the PBS-treated control (Figures 2E,F). Furthermore, while the percentages of CD4^+^IFN-γ^+^ (Th1) and CD4^+^Foxp3^+^ (Treg) cells remained unchanged, percentages of CD4^+^IL17^+^, CD4^+^GM-CSF^+^, and IFN-γ^+^IL-17^+^ cells decreased dramatically after carnosol treatment (*p* < 0.001; Figures 2G–K; Figure S1 in Supplementary Material). These results indicate that carnosol may play a significant role in the inhibition of CNS inflammatory infiltration, especially in the pathogenic Th17 cell population.

**Figure 3:**
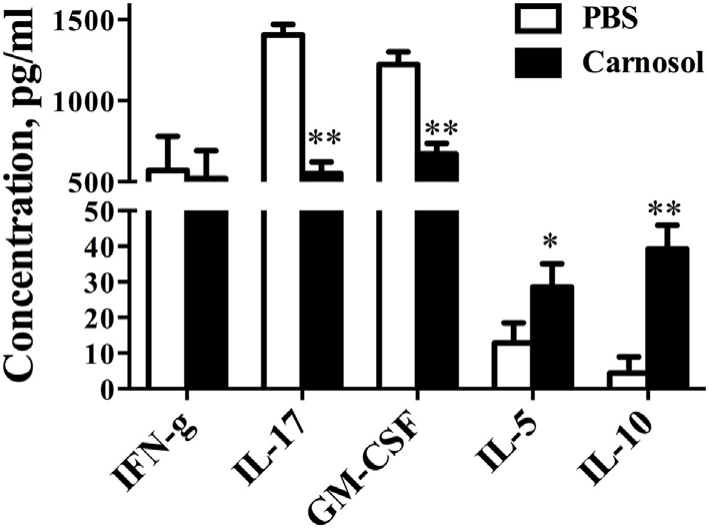
Carnosol treatment decreased inflammation and cytokine production. Mice were treated with PBS or carnosol at the day of experimental autoimmune encephalomyelitis induction and sacrificed at day 30 p.i. as described in Figure 1A. Splenocytes were harvested and stimulated with 25 µg/ml MOG_35–55_ for 3 days. Cytokine concentrations in culture supernatants were measured by ELISA. *n* = 5. Symbols represent mean ± SD (*n* = 5 each group). *p < 0.05 and **p < 0.01. Nonparametric test. One representative of three independent experiments is shown.

To study the autoantigen-induced cytokine production in the peripheral immune system of carnosol-treated mice, spleen cells were collected at day 30 p.i. and pulsed with MOG_35–55_. As shown in Figure 3, the protein levels of IL-17 and GM-CSF in cell culture supernatants were significantly decreased in the carnosol-treated group, which was consistent with the findings in the CNS infiltrated cells, as shown in Figures 2G–K. Overall, our data show that carnosol specifically inhibited the cytokine production of pathogenic Th17 cells.

### Carnosol Mediated Its Immunomodulation Function by Inhibiting Th17 Cell Differentiation

**Figure 4:**
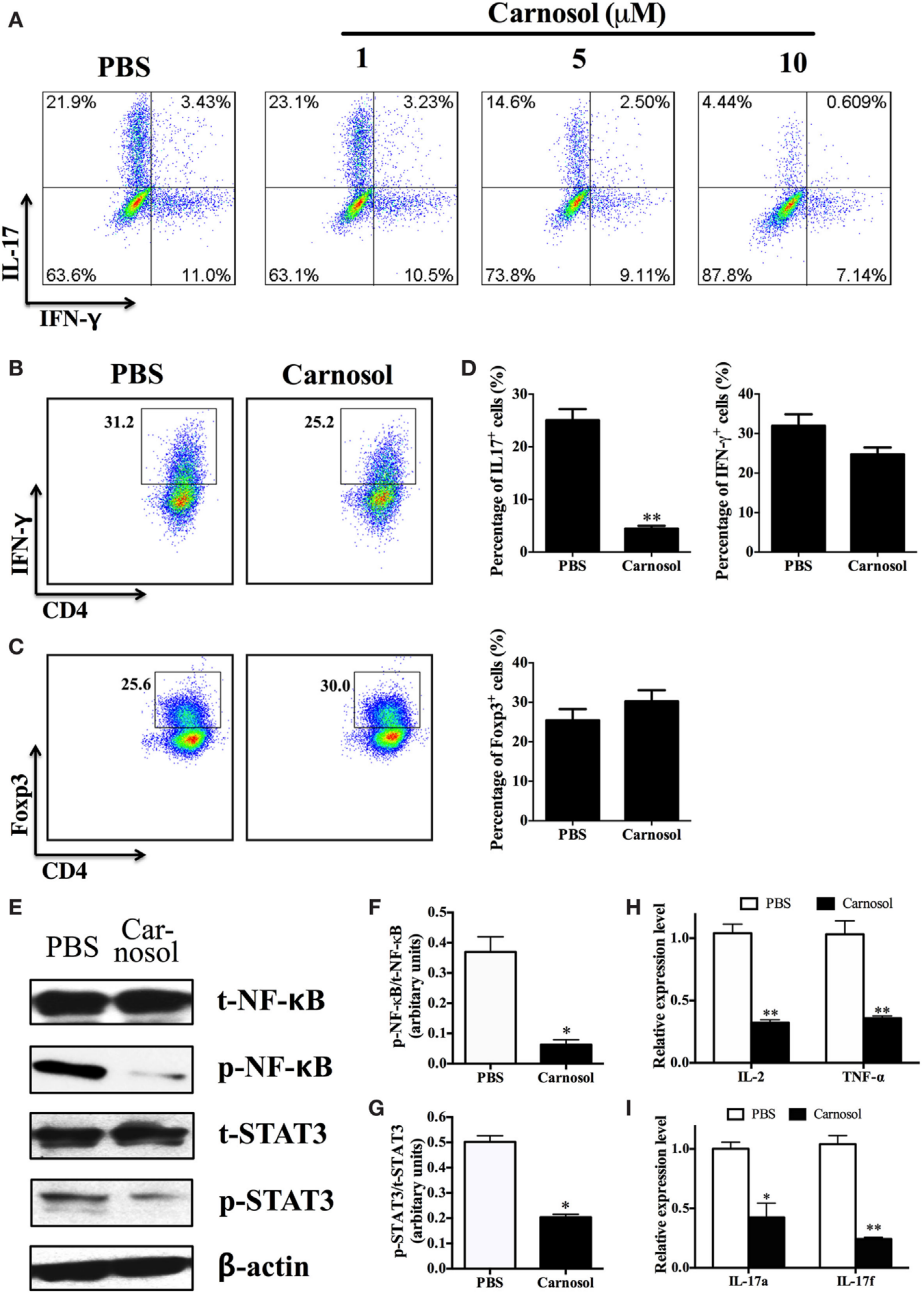
Carnosol suppressed Th17 cell differentiation by blocking the function of NF-κB and signal transducer and activator of transcription 3 (STAT3). **(A)** CD4^+^ cells were isolated from C57Bl/6 mice and cultured under the Th17 polarizing condition with different concentrations of carnosol for 3 days. Percentage of Th17 cells was analyzed by intracellular staining of IL-17. **(B,C)** CD4^+^ cells were cultured under the Th1 and regulatory T cell (Treg) polarizing condition with carnosol (10 µM) for 3 days. Percentages of Th1 and Treg cells were analyzed by intracellular staining of IFN-γ^+^ and Foxp3^+^, respectively. **(D)** Statistical analysis of **(A–C)**. **(E)** CD4^+^ T cells were cultured under Th17 polarizing condition and treated with 10 µM carnosol or PBS for 3 days. Cells were then analyzed for NF-κB and STAT3 expression by Western blot. **(F,G)** Statistical analysis of **(E)**. **(H,I)** Cells were harvested as described in **(E)** and subjected to RNA extraction and cDNA production. Expression of pro-inflammation cytokines and IL-17 members (IL-17a and IL-17f) was determined by real-time PCR. Symbols represent mean ± SD (*n* = 3 each group). *p < 0.05 and **p < 0.01. Student’s *t*-test. One representative of three independent experiments is shown.

To clarify the mechanism underlying the effects of carnosol on CD4^+^ T cell subsets, we defined its function in Th1, Th17, and Treg cell polarization *in vitro*. Under Th17-differentiation condition, about 25% of CD4^+^ cells were IL-17^+^ in the PBS group, while carnosol treatment at a dose of 10 µM significantly reduced Th17-polarized (IL-17-producing) CD4^+^ T cells (25.06 ± 2.13 vs. 4.47 ± 0.52%, *p* < 0.01) (Figures 4A,D). In addition, carnosol treatment suppressed Th17 differentiation in a dose-dependent manner. We then investigated the effects of carnosol on Th1 and Treg cell differentiation. In contrast to the findings for Th17 cells, IFN-γ or Foxp3 expression under Th1 or Treg polarizing condition was not significantly affected under carnosol treatment (Figures 4B–D). Taken together, these data suggest that carnosol selectively inhibits Th17 polarization.

### Carnosol Suppressed STAT3 and NF-κB Phosphorylation, Which Is Required for Th17 Differentiation

Inflammatory cytokine production depends on early events in the NF-κB signaling pathway (28). In order to study the mode of action of carnosol in T cell differentiation, the phosphorylation status of NF-κB was determined by Western blot. p65 phosphorylation at Ser536 regulates its activation and nuclear translocation (29). Results showed that carnosol suppressed cell response by a shift of NF-κBp65 to the cell nucleus, which was demonstrated by the proper shift in the ratio of phosphorylation NF-κB/total NF-κB (Figures 4E,F). Further, the pro-inflammatory cytokines in the downstream of NF-κB signal pathway, including IL-2 and TNF-α, were also significantly decreased (Figure 4H).

Signal transducer and activator of transcription 3 activities play an important role in the differentiation of Th17 cells. We determined that the basal STAT3 phosphorylation level was significantly decreased. The phosphorylation status at Tyr705 induced nuclear translocation and DNA binding, which promotes IL-17 production (30). Our results showed that carnosol treatment significantly suppressed STAT3 activation (Figures 4E,G) and IL-17A and IL-17F production of Th17 cells (Figure 4I) compared with the PBS-treated cells. In contrast, similar expression levels were observed for NF-κB and STAT4 phosphorylation in carnosol- and PBS-treated Th1 cells (Figure S2 in Supplementary Material). Together, these results indicate that carnosol may specifically inhibit differentiation of Th17 cells but not Th1 cells.

### Carnosol Suppressed Pathogenicity of Th17 Cells in Passive EAE

**Figure 5:**
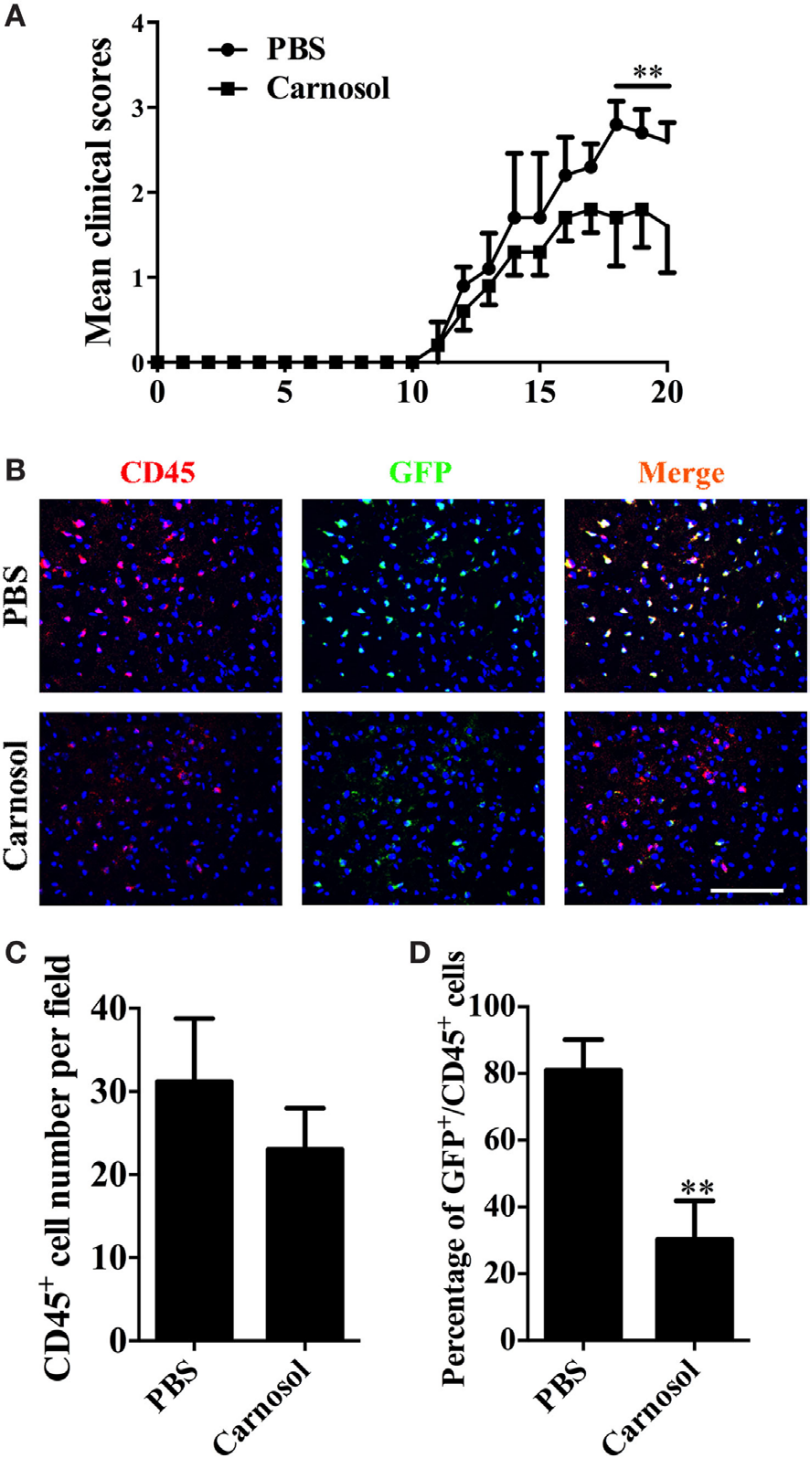
Carnosol decreased clinical severity in an adoptive transfer model of experimental autoimmune encephalomyelitis (EAE). For adoptive transfer EAE, single-cell suspensions were derived from spleen and lymph nodes of IL-17A-IRES-GFP EAE mice at day 10 p.i. MOG (25 µg/ml) plus IL-23 (10 ng/ml) and IL-2 (2 ng/ml) were added to cultures in the presence or absence of carnosol (10 µM) for 3 days. 1 × 10^6^ CD4 T cells were i.v. injected to the recipient mice. **(A)** Mean clinical score of adoptive transfer EAE (mean ± SD; *n* = 5 each group). **p < 0.01, Two-way ANOVA with Sidak test. **(B)** Mice were sacrificed at day 20 after cell transfer, and brains were subjected to immunostaining analysis of CD45^+^ and GFP^+^ cells (marker for Th17 cells). Statistical analyses of total CD45^+^ cell numbers **(C)** and the percentage of GFP^+^CD45^+^ cell **(D)** for staining in **(B)** are shown. Scale bar = 100 µm. Symbols represent mean ± SD (*n* = 5 each group) **p < 0.01, determined by two-way ANOVA **(A)**, or nonparametric test **(C,D)**. One representative of two independent experiments is shown.

To assess the effect of carnosol on the encephalitogenicity of Th17 cells, at day 10 p.i., MNCs were collected from lymph nodes and spleen of IL-17A-IRES-GFP mice (C57BL/6 background), of which IL-17A-producing cells are GFP^+^ (The Jackson Laboratory, Stock # 018472). Cells were cultured under Th17-polarizing conditions with PBS or carnosol, and stimulated by MOG_35–55_ (20 µg/ml). After 3 days of culture, CD4^+^ T cells were separated and i.v. injected into naïve C57BL/6 recipient mice. As shown in Figure 5A, carnosol-treated T cells transferred significantly reduced clinical disease compared to the PBS-treated group (*p* < 0.01). Mice were sacrificed after 20 days, and brain tissues from different groups were collected for immunohistochemistry. Results showed similar CD45^+^ cell numbers in the tissue; however, in the CNS, the percentages of GFP^+^/CD45^+^ cells in the carnosol-treated group were markedly reduced compared with the PBS-treated group (*p* < 0.01; Figures 5B–D). These *in vivo* results further demonstrated a suppression function of carnosol on the encephalitogenicity of MOG-reactive Th17 cells.

### Carnosol Alleviated Clinical Disease When Treatment Started at Chronic Stage of EAE

**Figure 6:**
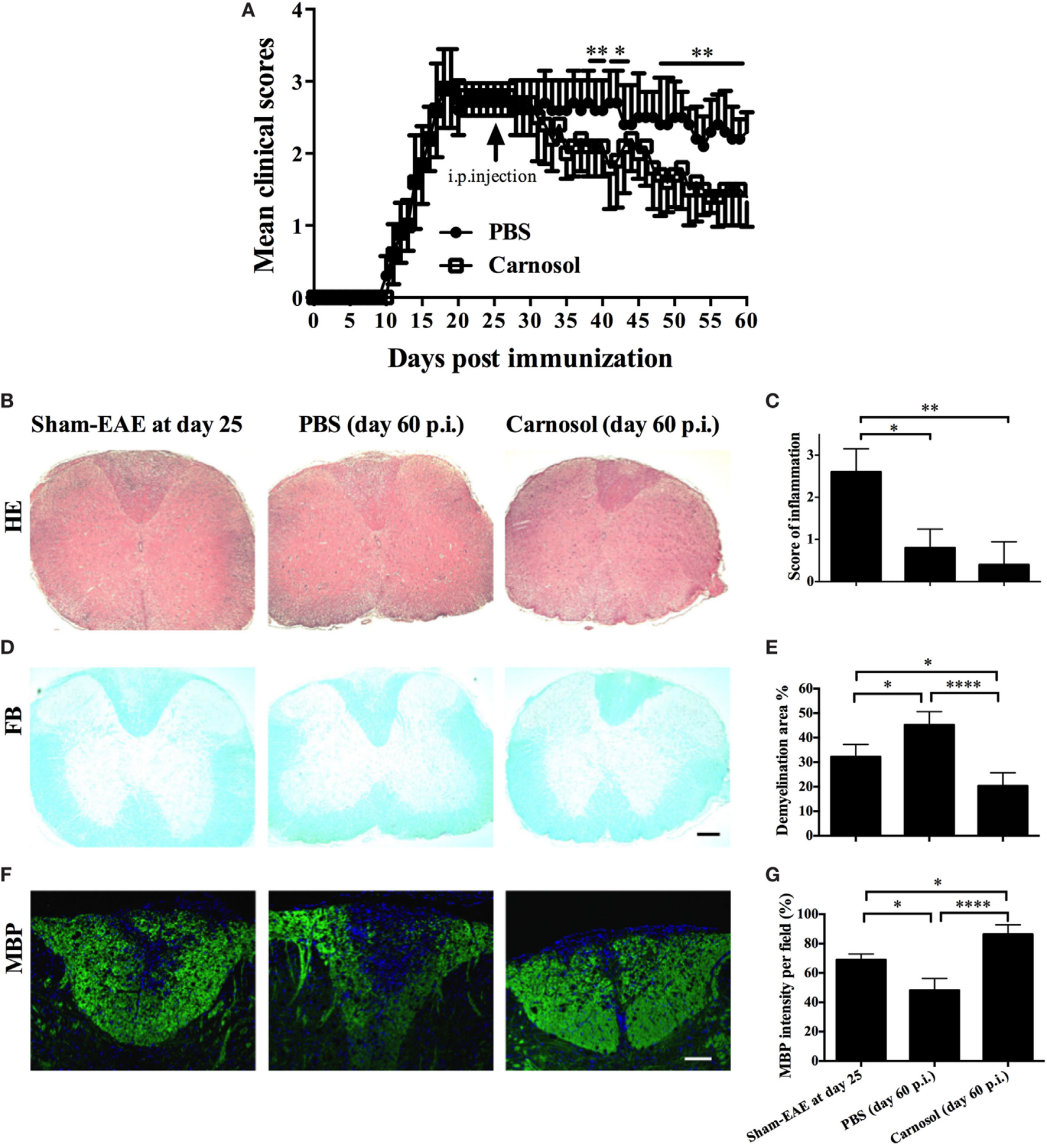
Carnosol treatment alleviated the clinical severity of chronic experimental autoimmune encephalomyelitis (EAE) mice. **(A)** Clinical scores of carnosol- and PBS-treated mice at the chronic stage (treatment starting from day 25 p.i.) of EAE. Mice were sacrificed at day 60 p.i. (*n* = 5 each group), and spinal cords were harvested and evaluated for cell infiltration by H&E staining **(B)**, which was scored on a 0–3 scale **(C)**, and for demyelination by Luxol fast blue **(D)**. **(E)** Demyelination area was measured using Image-Pro Plus software. **(F)** Sections of lumbar spinal cord from **(A)** were assayed for demyelination by MBP staining. **(G)** Quantitative analysis of MBP expression. MBP intensity was measured in the lesion areas in the lumbar spinal cord using Image-Pro. Data represent mean ± SD (*n* = 10 each group). Scale bar = 1 mm **(B,D)** or 100 µm **(F)**. *p < 0.05, **p < 0.01, and ****p < 0.0001. Student’s *t*-test. One representative of three independent experiments is shown.

To further explore the therapeutic effects of carnosol, the chronic EAE model was used in this study. Mice were treated starting from day 25 p.i., when CNS demyelination and chronic tissue damage were already established. While clinical scores in the PBS-injected mice remained at 2.5–3.0, the disease was significantly alleviated in the carnosol-treated group after 10 days of treatment (*p* < 0.01–0.001; Figure 6A). The results indicate that, compared to the PBS-treated mice, carnosol showed potential for blockade of demyelination and recovery from neurological damage in the CNS, even when treatment was started after the peak of disease.

Compared to acute EAE (e.g., day 25 p.i.), in chronic EAE (e.g., day 60 p.i.; Figures 6B,C), rare infiltration inflammation cells were observed in the white matter of both PBS- and carnosol-treated mice, suggesting that neuroinflammation is no longer the major pathogenesis in the chronic stage (23). On the other hand, while PBS-treated EAE mice tended to have more severe demyelination, as shown by LFB and MBP staining, the demyelination area was obviously decreased in carnosol-treated mice compared to PBS-treated control mice. Increased MBP expression after carnosol treatment compared to that before treatment (day 25 p.i.) suggests that carnosol might induce myelin protein regeneration (Figures 6D–G).

### Carnosol Promoted an M1/M2 Phenotype Shift of Macrophage/Microglia

**Figure 7:**
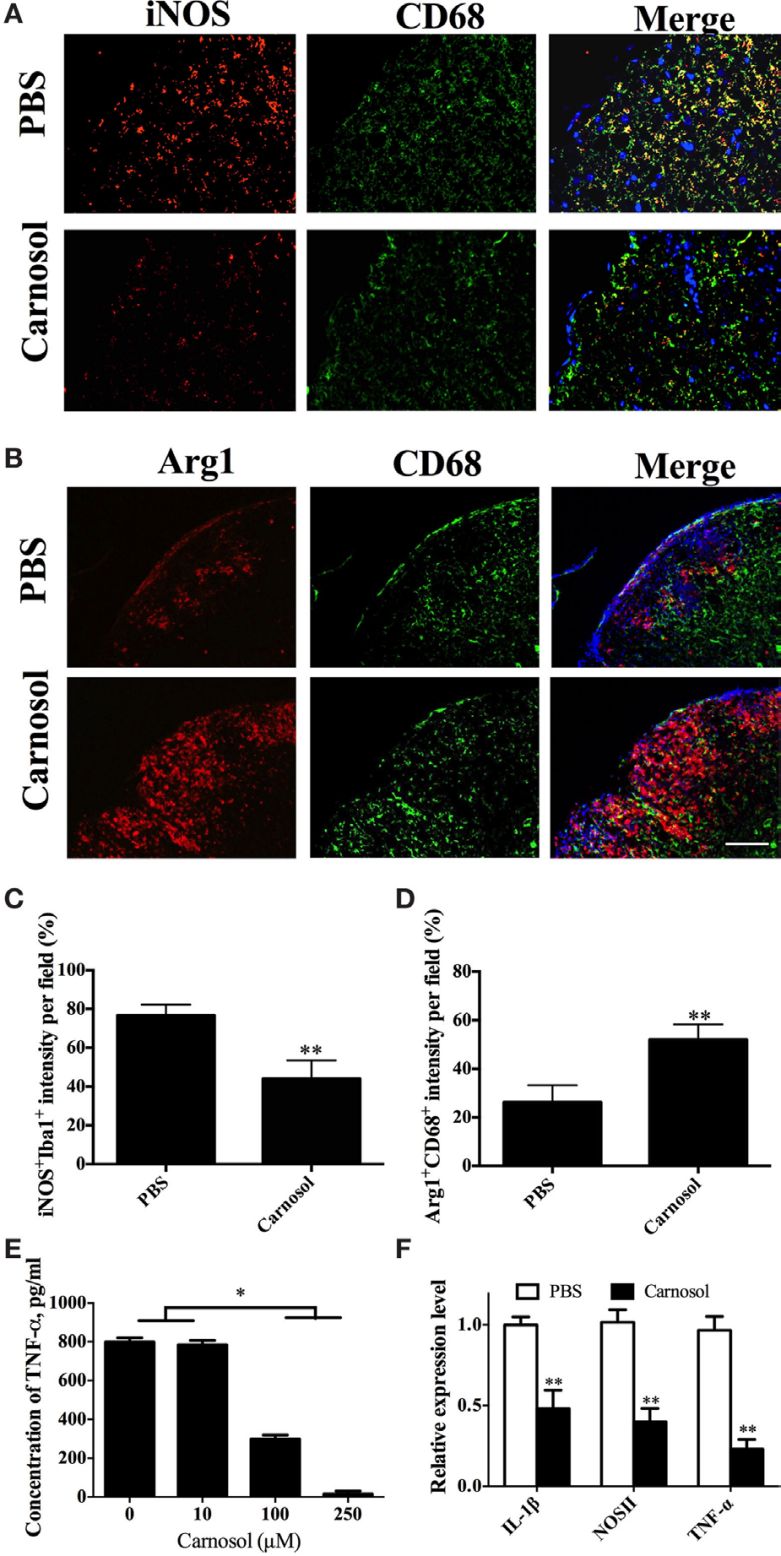
Carnosol promoted an M2 phenotype in macrophages/microglia. Spinal cords of mice described in Figure 6 were stained for markers for M1 [iNOS; **(A)**] and M2 [Arg-1; **(B)**] on microglia/infiltrating macrophages (CD68^+^ cells). **(C,D)** Quantitative analysis of the percentages of double positive cells. **(E,F)** Primary microglia were prepared from newborn B6 mice, stimulated with LPS (100 ng/ml), and treated with carnosol at different concentrations for 2 days and **(E)** supernatants were harvested for TNF-α production and **(F)** cells were collected for expression levels of IL-1β, NOSII, and TNF-α by real-time PCR. Glyceraldehyde 3-phosphate dehydrogenase was used as an internal control. Scale bar = 100 µm. Data are shown as mean values ± SD (*n* = 5 each group). ANOVA with Tukey’s multiple comparisons test was used. *p < 0.05 and **p < 0.01. One representative of three independent experiments is shown.

Given that microglia/infiltrating macrophages with the activated type 1 phenotype (M1) have a significant role in CNS inflammation during EAE chronicity, whereas type 2 phenotype (M2) cells are immunomodulatory and promyelinating (31, 32), we determined the effects of carnosol on these cells in the CNS tissues of EAE mice that were euthanized after 60 days p.i. The number of M1 microglia/infiltrating macrophages (iNOS^+^CD68^+^) was decreased and an increase in M2 (Arg1^+^CD68^+^) phenotype was observed in carnosol-treated mice compared to PBS-treated control (Figures 7A–D). These results indicated that, at least partially, carnosol inhibited demyelination and promoted myelin recovery through inhibiting M1 microglia and switching them to M2. To further confirm this hypothesis, primary microglia were cultured with or without carnosol. Carnosol effectively inhibited production of important mediators of microglia activation, e.g., TNF-α (Figure 7E), and expression levels of IL-1β, NOSII, and TNF-α were also significantly decreased (Figure 7F). These results indicated that carnosol inhibits the infiltration of M1 phenotype microglia and switches it to a promyelinating and immunoregulatory M2 phenotype that promotes the process of myelin regeneration (32).

## Discussion

This work for the first time shows the beneficial effect of carnosol on both acute and chronic stages of EAE. Carnosol significantly decreased inflammatory infiltration into the CNS and the demyelination process, thus halting disease development. The role of carnosol in acute EAE is primarily due to its inhibitory effect on Th17 cell differentiation, CNS infiltration, and encephalitogenicity, in which the STAT3 signaling pathway plays an important role. Further, the shift of microglia/infiltrated macrophage phenotype from a pro-inflammatory (M1) to an immunoregulatory one (M2) may be an important mechanism underlying the therapeutic effect of carnosol on the chronic stage of EAE.

Carnosol, an ortho-diphenolic of abietane-type diterpene-lactone, consists of an abietane carbon skeleton with hydroxyl groups at positions C-11 and C-12 and a lactone moiety across the B ring (18). Carnosol showed a broad range of physiological benefits and bio-pharmacological effects, as well as exerted strong anti-oxidant, anti-cancer, and neuroprotection effects (17, 20). Furthermore, carnosol was reported to exert anti-inflammatory effects by reducing cytokine release (e.g., IL-1, IL-6) and iNOS formation (18). Also, carnosol, as an anti-inflammatory and anti-oxidant agent, has been considered as a potentially promising therapeutic drug for many incurable diseases, such as neurodegeneration, cancer, and cardiovascular disorders (33, 34). However, the mechanism underlying these functions has not been completely elucidated. Although it has already been shown that carnosol stimulates the MAPKs signaling pathway and down-regulates multiple transcription factors, including NF-κB as well as pro-inflammation protein such as COX-2 level (35–37), to our knowledge, this is the first study to show that carnosol treatment leads to an inhibition in Th17 differentiation and that it modulates microglial switch.

The major challenge for the clinical application of natural compounds is determining their detailed molecular mechanism (4). Indeed, the mechanism of carnosol’s action on T helper cell differentiation in autoimmune disease remains largely unknown. It has been suggested that carnosol suppresses inflammation by targeting NF-κB signaling (37, 38), whose activation has been found in MS brain lesions (39, 40) and peripheral blood (41), as well as in the development of EAE (42, 43). Further, IL-17 plays a key role in the pathogenesis of MS and EAE (9, 44). Specifically, activated STAT3 is considered to be necessary for IL-17 production in mouse and human Th17 cells (45, 46). STAT3 controls various genes that contribute to the Th17 population cells including the IL-17 locus itself (47), and binds to genes encoding transcription factors that are critical for Th17 polarization, including Rorc, Irf4, and Batf (48). In our study, carnosol altered the level of Th17 lineage-associated cytokine IL-17. This finding suggests that carnosol inhibits polarization of T cells into Th17 cells, which may be due to carnosol’s ability to diminish Th17-associated cytokines by targeting the NF-κB signaling pathway. In response to cytokines, STAT3 is phosphorylated by receptor-associated Janus kinases and forms homo- or heterodimers that translocate to the cell nucleus, where they act as transcription activators. Here, we show that carnosol suppressed STAT3 phosphorylation at the site of tyrosine 705, in response to the ligand IL-6. These findings further identified the mechanism of carnosol through suppressed NF-κB and STAT3 phosphorylation to block Th17 differentiation.

We have further identified the therapeutic effects of carnosol on chronic stage of EAE, and investigated the involvement of M1/M2 microglia shift as a potential mechanism of its action. Persistent CNS inflammation, particularly the activation of infiltrated macrophage/microglia, is recognized to be a crucial mechanism underlying EAE chronicity (49). Pro-inflammatory cytokines, including IL-1β, IL-6, and TNF-α, were secreted by these inflammatory cells, which, together with the accumulation of neurodegeneration inhibitors, form a hostile microenvironment against remyelination and neural repair (24). Therefore, diminishing the inflammatory cytokines of the CNS niche and promoting its change to a supportive environment for neural repair and remyelination will be helpful for treatment. Here, we showed that carnosol suppressed infiltrated macrophage/microglia activation both in EAE mice *in vivo* and microglia culture *in vitro*. A shift from M1 to M2 phenotype was observed following carnosol treatment. Previous studies indicated that carnosol reduced LPS-induced iNOS mRNA and protein expression. Administration of carnosol resulted in a reduction of nuclear factor-kappa B (NF-κB) subunit translocation and NF-κB DNA binding activity in activated macrophages (50). Further experimental data added proof that carnosol blockades the IL-1β induced nuclear translocation of NF-κBp65, indicating that it mainly regulates through the NF-κB signaling (38). These findings were consistent with our results and indicated that carnosol could switch infiltrated macrophages/microglia from M1 to M2 phenotype and may play an essential role in myelin protein recovery.

One of the major mechanisms contributing to the chronic progression in MS is loss of neurotrophic factor support for both oligodendrocytes and neurons, resulting in persistent damage to CNS tissue damage, i.e., demyelination, axonal degeneration, and neuronal dysfunction (23). Exploring a novel medicine that both targets neuroinflammation and promotes neuroregeneration will, therefore, be of great value. Recently, Wang et al. showed the protective role of carnosol against spinal cord injury (37). This study led us to determine whether carnosol has a neuroprotective function in demyelinating disease. In the present study, we observed that carnosol blocks demyelination by means of the M1/M2 switch. However, no significant differences were observed in OPC differentiation *in vitro* or in the cuprizone-induced demyelination model (data not shown). This finding may illustrate that the underlying mechanism of carnosol-induced recovery in EAE mice is not due to its direct effect on oligodendrocyte differentiation/maturation, but rather an indirect effect through immunomodulation and reduced CNS inflammation and the M1/M2 switch, thus providing a supportive microenvironment for neural cells.

Although we demonstrated the efficacy of carnosol treatment of EAE, the immunomodulatory mechanism is not clear. We showed that carnosol could suppress IL-17 and GM-CSF production of splenocytes, but we also found that carnosol exerts its anti-inflammatory effect on microglia. Increasing evidence shows that carnosol can cross the blood–brain barrier (BBB) as a neuroprotective agent. We, therefore, provide compelling evidence supporting an effective role of carnosol in inhibiting Th17 cell differentiation in the periphery and modulating microglia phenotype by penetrating the BBB in the CNS.

In addition, a previous study showed that carnosol has anti-tumor capacity through prevention of Treg cell differentiation, decreasing IL-4 and IL-10 production, and enhancing IFN-γ secretion in tumor-associated lymphocyte populations (51). Tumor Tregs are a highly heterogeneous population that arises through disparate pathways and mediates immunologic effects by various means including soluble cytokines (52). An explanation of the principal mechanism of their increase would include a reaction to autoimmunity, tumor-specific factors, and control of inflammation. Although autoimmune disease and cancer both arise from dysfunctions in the immune system, these dysfunctions are extremely different. This phenomenon depends on the complex *in vivo* process and is also due to the different molecule target. Although it appears paradoxical that carnosol has anti-tumor capacity and the ability to suppress EAE, we hypothesize that as a small molecule, carnosol may bind to various molecular sites and induce different signaling pathways, an ability that may be determined by different microenvironments.

In summary, the present study demonstrates that carnosol ameliorated clinical severity of acute and chronic EAE. We propose that these effects are due to the inhibition of Th17 cell polarization and a remarkably switched phenotype of infiltrated macrophages and activated microglia. Taken together, our data indicate that carnosol is a natural molecule that has potential for the treatment of MS, and likely for autoimmune diseases in general.

## Ethics Statement

This study was carried out in accordance with the recommendations of Institutional Animal Care and Use guidelines, Institutional Animal Care and Use Committee of Shaanxi Normal University. The protocol was approved by the Institutional Animal Care and Use Committee of Shaanxi Normal University.

## Author Contributions

XL and YZ conceived and designed the experiments, and wrote the manuscript. XL, LZ, FZ, J-JH, and SL carried out the experiments. LZ, Z-ZW, and G-XZ helped to design the experiments and analyzed data. All authors read and approved the final manuscript. We thank Katherine Regan for editorial assistance.

## Conflict of Interest Statement

The authors declare that the research was conducted in the absence of any commercial or financial relationships that could be construed as a potential conflict of interest.

## References

[1] Yang CL Or TC Ho MH Lau AS. Scientific basis of botanical medicine as alternative remedies for rheumatoid arthritis. Clin Rev Allergy Immunol (2013) 44(3):284–300. 10.1007/s12016-012-8329-8 22700248

[2] Lassmann H Bradl M. Multiple sclerosis: experimental models and reality. Acta Neuropathol (2017) 133(2):223–44. 10.1007/s00401-016-1631-4 27766432 PMC5250666

[3] Yshii LM Hohlfeld R Liblau RS. Inflammatory CNS disease caused by immune checkpoint inhibitors: status and perspectives. Nat Rev Neurol (2017) 13(12):755–63. 10.1038/nrneurol.2017.144 29104289

[4] Zhang Y Li X Ciric B Ma CG Gran B Rostami A Therapeutic effect of baicalin on experimental autoimmune encephalomyelitis is mediated by SOCS3 regulatory pathway. Sci Rep (2015) 5:17407. 10.1038/srep17407 26616302 PMC4663791

[5] El-Behi M Ciric B Dai H Yan Y Cullimore M Safavi F The encephalitogenicity of T(H)17 cells is dependent on IL-1- and IL-23-induced production of the cytokine GM-CSF. Nat Immunol (2011) 12(6):568–75. 10.1038/ni.2031 21516111 PMC3116521

[6] Annunziato F Romagnani C Romagnani S. The 3 major types of innate and adaptive cell-mediated effector immunity. J Allergy Clin Immunol (2015) 135(3):626–35. 10.1016/j.jaci.2014.11.001 25528359

[7] Sutton CE Lalor SJ Sweeney CM Brereton CF Lavelle EC Mills KH. Interleukin-1 and IL-23 induce innate IL-17 production from gammadelta T cells, amplifying Th17 responses and autoimmunity. Immunity (2009) 31(2):331–41. 10.1016/j.immuni.2009.08.001 19682929

[8] Oukka M. Interplay between pathogenic Th17 and regulatory T cells. Ann Rheum Dis (2007) 66(Suppl 3):iii87–90. 10.1136/ard.2007.078527 17934104 PMC2095282

[9] Xiao X Shi X Fan Y Wu C Zhang X Minze L The costimulatory receptor OX40 inhibits interleukin-17 expression through activation of repressive chromatin remodeling pathways. Immunity (2016) 44(6):1271–83. 10.1016/j.immuni.2016.05.013 27317259 PMC4917494

[10] Zhao M Tan Y Peng Q Huang C Guo Y Liang G IL-6/STAT3 pathway induced deficiency of RFX1 contributes to Th17-dependent autoimmune diseases via epigenetic regulation. Nat Commun (2018) 9(1):583. 10.1038/s41467-018-02890-0 29422534 PMC5805701

[11] Filippini G Del Giovane C Clerico M Beiki O Mattoscio M Piazza F Treatment with disease-modifying drugs for people with a first clinical attack suggestive of multiple sclerosis. Cochrane Database Syst Rev (2017) 4:Cd012200. 10.1002/14651858.CD012200.pub2 28440858 PMC6478290

[12] Hemmer B Muhlau M Multiple sclerosis in 2016: immune-directed therapies in MS – efficacy and limitations. Nat Rev Neurol (2017) 13(2):72–4. 10.1038/nrneurol.2017.2 28106067

[13] Wei CB Tao K Jiang R Zhou LD Zhang QH Yuan CS Quercetin protects mouse liver against triptolide-induced hepatic injury by restoring Th17/Treg balance through Tim-3 and TLR4-MyD88-NF-kappaB pathway. Int Immunopharmacol (2017) 53:73–82. 10.1016/j.intimp.2017.09.026 29040945

[14] Thome R de Carvalho AC Alves da Costa T Ishikawa LL Fraga-Silva TF Sartori A Artesunate ameliorates experimental autoimmune encephalomyelitis by inhibiting leukocyte migration to the central nervous system. CNS Neurosci Ther (2016) 22(8):707–14. 10.1111/cns.12561 27165523 PMC6492826

[15] Kan QC Zhang HJ Zhang Y Li X Xu YM Thome R Matrine treatment blocks NogoA-induced neural inhibitory signaling pathway in ongoing experimental autoimmune encephalomyelitis. Mol Neurobiol (2017) 54(10):8404–18. 10.1007/s12035-016-0333-1 27933584

[16] Liu M Zhou X Zhou L Liu Z Yuan J Cheng J Carnosic acid inhibits inflammation response and joint destruction on osteoclasts, fibroblast-like synoviocytes, and collagen-induced arthritis rats. J Cell Physiol (2018) 233(8):6291–303. 10.1002/jcp.26517 29521424

[17] Bendif H Boudjeniba M Djamel Miara M Biqiku L Bramucci M Caprioli G *Rosmarinus eriocalyx*: an alternative to *Rosmarinus officinalis* as a source of antioxidant compounds. Food Chem (2017) 218:78–88. 10.1016/j.foodchem.2016.09.063 27719960

[18] Kashyap D Kumar G Sharma A Sak K Tuli HS Mukherjee TK. Mechanistic insight into carnosol-mediated pharmacological effects: recent trends and advancements. Life Sci (2017) 169:27–36. 10.1016/j.lfs.2016.11.013 27871947

[19] Oliviero F Scanu A Zamudio-Cuevas Y Punzi L Spinella P Anti-inflammatory effects of polyphenols in arthritis. J Sci Food Agri (2017) 98(5):1653–9. 10.1002/jsfa.8664 28886220

[20] de Oliveira MR. The dietary components carnosic acid and carnosol as neuroprotective agents: a mechanistic view. Mol Neurobiol (2016) 53(9):6155–68. 10.1007/s12035-015-9519-1 26553346

[21] Samarghandian S Borji A Farkhondeh T. Evaluation of antidiabetic activity of carnosol (phenolic diterpene in Rosemary) in streptozotocin-induced diabetic rats. Cardiovasc Hematol Disord Drug Targets (2017) 17(1):11–7. 10.2174/1871529X16666161229154910 28034282

[22] Ishida Y Yamasaki M Yukizaki C Nishiyama K Tsubouchi H Okayama A Carnosol, rosemary ingredient, induces apoptosis in adult T-cell leukemia/lymphoma cells via glutathione depletion: proteomic approach using fluorescent two-dimensional differential gel electrophoresis. Human Cell (2014) 27(2):68–77. 10.1007/s13577-013-0083-6 24323765

[23] Li X Zhang Y Yan Y Ciric B Ma CG Gran B Neural stem cells engineered to express three therapeutic factors mediate recovery from chronic stage CNS autoimmunity. Mol Ther (2016) 24(8):1456–69. 10.1038/mt.2016.104 27203442 PMC5023377

[24] Li X Zhang Y Yan Y Ciric B Ma CG Chin J LINGO-1-Fc-transduced neural stem cells are effective therapy for chronic stage experimental autoimmune encephalomyelitis. Mol Neurobiol (2017) 54(6):4365–78. 10.1007/s12035-016-9994-z 27344330

[25] Zhang Y Han JJ Liang XY Zhao L Zhang F Rasouli J miR-23b suppresses leukocyte migration and pathogenesis of experimental autoimmune encephalomyelitis by targeting CCL7. Mol Ther (2018) 26(2):582–92. 10.1016/j.ymthe.2017.11.013 29275848 PMC5835026

[26] Zhao L Li X Ye ZQ Zhang F Han JJ Yang T Nutshell extracts of *Xanthoceras sorbifolia*: a new potential source of bioactive phenolic compounds as a natural antioxidant and immunomodulator. J Agric Food Chem (2018) 66(15):3783–92. 10.1021/acs.jafc.7b05590 29613792

[27] Wang LM Zhang Y Li X Zhang ML Zhu L Zhang GX Nr4a1 plays a crucial modulatory role in Th1/Th17 cell responses and CNS autoimmunity. Brain Behav Immun (2018) 68:44–55. 10.1016/j.bbi.2017.09.015 28962999

[28] Visekruna A Volkov A Steinhoff U A key role for NF-kappaB transcription factor c-Rel in T-lymphocyte-differentiation and effector functions. Clin Dev Immunol (2012) 2012:239368 10.1155/2012/239368 22481964 PMC3310234

[29] Sasaki CY Barberi TJ Ghosh P Longo DL. Phosphorylation of RelA/p65 on serine 536 defines an I{kappa}B{alpha}-independent NF-{kappa}B pathway. J Biol Chem (2005) 280(41):34538–47. 10.1074/jbc.M504943200 16105840

[30] Chaudhry A Rudra D Treuting P Samstein RM Liang Y Kas A CD4+ regulatory T cells control TH17 responses in a Stat3-dependent manner. Science (2009) 326(5955):986–91. 10.1126/science.1172702 19797626 PMC4408196

[31] Pluchino S Muzio L Imitola J Deleidi M Alfaro-Cervello C Salani G Persistent inflammation alters the function of the endogenous brain stem cell compartment. Brain (2008) 131(Pt 10):2564–78. 10.1093/brain/awn198 18757884 PMC2570715

[32] Miron VE Boyd A Zhao JW Yuen TJ Ruckh JM Shadrach JL M2 microglia and macrophages drive oligodendrocyte differentiation during CNS remyelination. Nat Neurosci (2013) 16(9):1211–8. 10.1038/nn.3469 23872599 PMC3977045

[33] Foresti R Bains SK Pitchumony TS de Castro Bras LE Drago F Dubois-Rande JL Small molecule activators of the Nrf2-HO-1 antioxidant axis modulate heme metabolism and inflammation in BV2 microglia cells. Pharmacol Res (2013) 76:132–48. 10.1016/j.phrs.2013.07.010 23942037

[34] Giacomelli C Daniele S Natali L Iofrida C Flamini G Carnosol controls the human glioblastoma stemness features through the epithelial-mesenchymal transition modulation and the induction of cancer stem cell apoptosis. Sci Rep (2017) 7(1):15174 10.1038/s41598-017-15360-2 29123181 PMC5680298

[35] Chen CC Chen HL Hsieh CW Yang YL Wung BS. Upregulation of NF-E2-related factor-2-dependent glutathione by carnosol provokes a cytoprotective response and enhances cell survival. Acta Pharmacol Sin (2011) 32(1):62–9. 10.1038/aps.2010.181 21151161 PMC4003314

[36] Martin D Rojo AI Salinas M Diaz R Gallardo G Alam J Regulation of heme oxygenase-1 expression through the phosphatidylinositol 3-kinase/Akt pathway and the Nrf2 transcription factor in response to the antioxidant phytochemical carnosol. J Biol Chem (2004) 279(10):8919–29. 10.1074/jbc.M309660200 14688281

[37] Wang ZH Xie YX Zhang JW Qiu XH Cheng AB Tian L Carnosol protects against spinal cord injury through Nrf-2 upregulation. J Recept Signal Transduct Res (2016) 36(1):72–8. 10.3109/10799893.2015.1049358 26791582

[38] Schwager J Richard N Fowler A Seifert N Raederstorff D. Carnosol and related substances modulate chemokine and cytokine production in macrophages and chondrocytes. Molecules (2016) 21(4):465. 10.3390/molecules21040465 27070563 PMC6274263

[39] Gveric D Kaltschmidt C Cuzner ML Newcombe J. Transcription factor NF-kappaB and inhibitor I kappaBalpha are localized in macrophages in active multiple sclerosis lesions. J Neuropathol Exp Neurol (1998) 57(2):168–78. 10.1097/00005072-199802000-00008 9600209

[40] Bonetti B Stegagno C Cannella B Rizzuto N Moretto G Raine CS. Activation of NF-kappaB and c-jun transcription factors in multiple sclerosis lesions. Implications for oligodendrocyte pathology. Am J Pathol (1999) 155(5):1433–8. 10.1016/S0002-9440(10)65456-9 10550297 PMC1866971

[41] Miterski B Bohringer S Klein W Sindern E Haupts M Schimrigk S Inhibitors in the NFkappaB cascade comprise prime candidate genes predisposing to multiple sclerosis, especially in selected combinations. Genes Immun (2002) 3(4):211–9. 10.1038/sj.gene.6363846 12058256

[42] Hucke S Eschborn M Liebmann M Herold M Freise N Engbers A Sodium chloride promotes pro-inflammatory macrophage polarization thereby aggravating CNS autoimmunity. J Autoimmun (2016) 67:90–101. 10.1016/j.jaut.2015.11.001 26584738

[43] Zhu S Pan W Song X Liu Y Shao X Tang Y The microRNA miR-23b suppresses IL-17-associated autoimmune inflammation by targeting TAB 2, TAB 3 and IKK-alpha. Nat Med (2012) 18(7):1077–86. 10.1038/nm.2815 22660635

[44] Patel DD Kuchroo VK. Th17 cell pathway in human immunity: lessons from genetics and therapeutic interventions. Immunity (2015) 43(6):1040–51. 10.1016/j.immuni.2015.12.003 26682981

[45] Chen Z Laurence A Kanno Y Pacher-Zavisin M Zhu BM Tato C Selective regulatory function of Socs3 in the formation of IL-17-secreting T cells. Proc Natl Acad Sci U S A (2006) 103(21):8137–42. 10.1073/pnas.0600666103 16698929 PMC1459629

[46] Mathur AN Chang HC Zisoulis DG Stritesky GL Yu Q O’Malley JT Stat3 and Stat4 direct development of IL-17-secreting Th cells. J Immunol (2007) 178(8):4901–7. 10.4049/jimmunol.178.8.4901 17404271

[47] Yang XP Ghoreschi K Steward-Tharp SM Rodriguez-Canales J Zhu J Grainger JR Opposing regulation of the locus encoding IL-17 through direct, reciprocal actions of STAT3 and STAT5. Nat Immunol (2011) 12(3):247–54. 10.1038/ni.1995 21278738 PMC3182404

[48] Durant L Watford WT Ramos HL Laurence A Vahedi G Wei L Diverse targets of the transcription factor STAT3 contribute to T cell pathogenicity and homeostasis. Immunity (2010) 32(5):605–15. 10.1016/j.immuni.2010.05.003 20493732 PMC3148263

[49] Rasmussen S Wang Y Kivisakk P Bronson RT Meyer M Imitola J Persistent activation of microglia is associated with neuronal dysfunction of callosal projecting pathways and multiple sclerosis-like lesions in relapsing – remitting experimental autoimmune encephalomyelitis. Brain (2007) 130(Pt 11):2816–29. 10.1093/brain/awm219 17890734

[50] Lo AH Liang YC Lin-Shiau SY Ho CT Lin JK. Carnosol, an antioxidant in rosemary, suppresses inducible nitric oxide synthase through down-regulating nuclear factor-kappaB in mouse macrophages. Carcinogenesis (2002) 23(6):983–91. 10.1093/carcin/23.6.983 12082020

[51] Rahnama M Mahmoudi M Zamani Taghizadeh Rabe S Balali-Mood M Karimi G Tabasi N Evaluation of anti-cancer and immunomodulatory effects of carnosol in a Balb/c WEHI-164 fibrosarcoma model. J Immunotoxicol (2015) 12(3):231–8. 10.3109/1547691X.2014.934975 25027673

[52] Curiel TJ. Regulatory T cells and treatment of cancer. Curr Opin Immunol (2008) 20(2):241–6. 10.1016/j.coi.2008.04.008 18508251 PMC3319305

