# Supplementary material for: Carnosol Modulates Th17 Cell Differentiation and Microglial Switch in Experimental Autoimmune Encephalomyelitis
Source: Front Immunol. 2018 Aug 13;9:1807. doi: 10.3389/fimmu.2018.01807 (PMC6100297; doi:10.3389/fimmu.2018.01807)
Supplement: Supplementary file 1 [file Data_Sheet_1.docx]

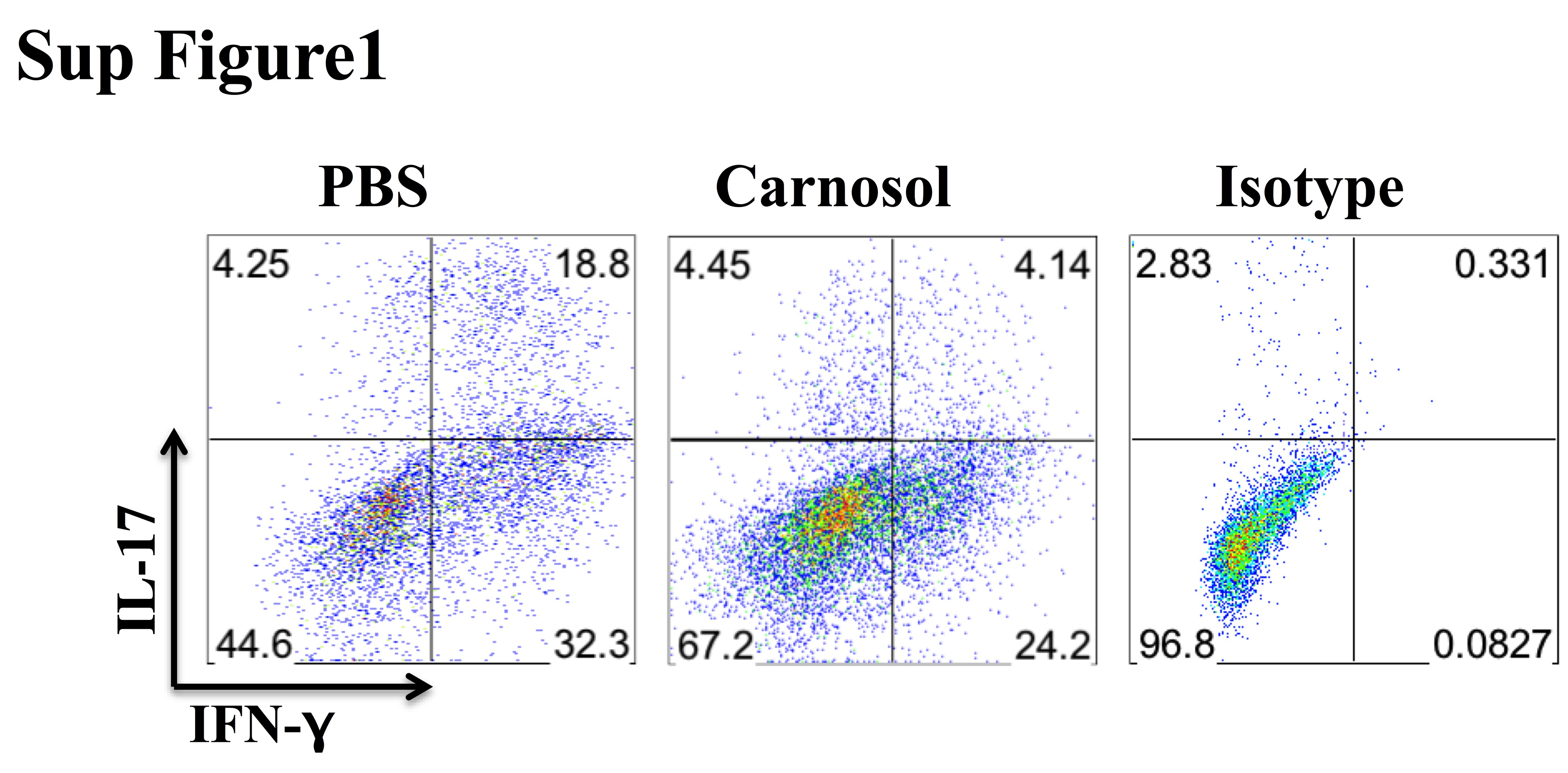


**Sup. Fig.1. Carnosol treatment reduced the proportion of IFN-γ^+^IL-17^+^ CD4^+^  T cells in the CNS.** Mice were treated with PBS or carnosol at the day of EAE induction and sacrificed at day 30 p.i. Spinal cords and brains were harvested and MNCs isolated (n = 10 each group). Frequencies of IFN-γ^+^IL-17^+^ cells among CD4^+^ cells were assessed by flow cytometry.


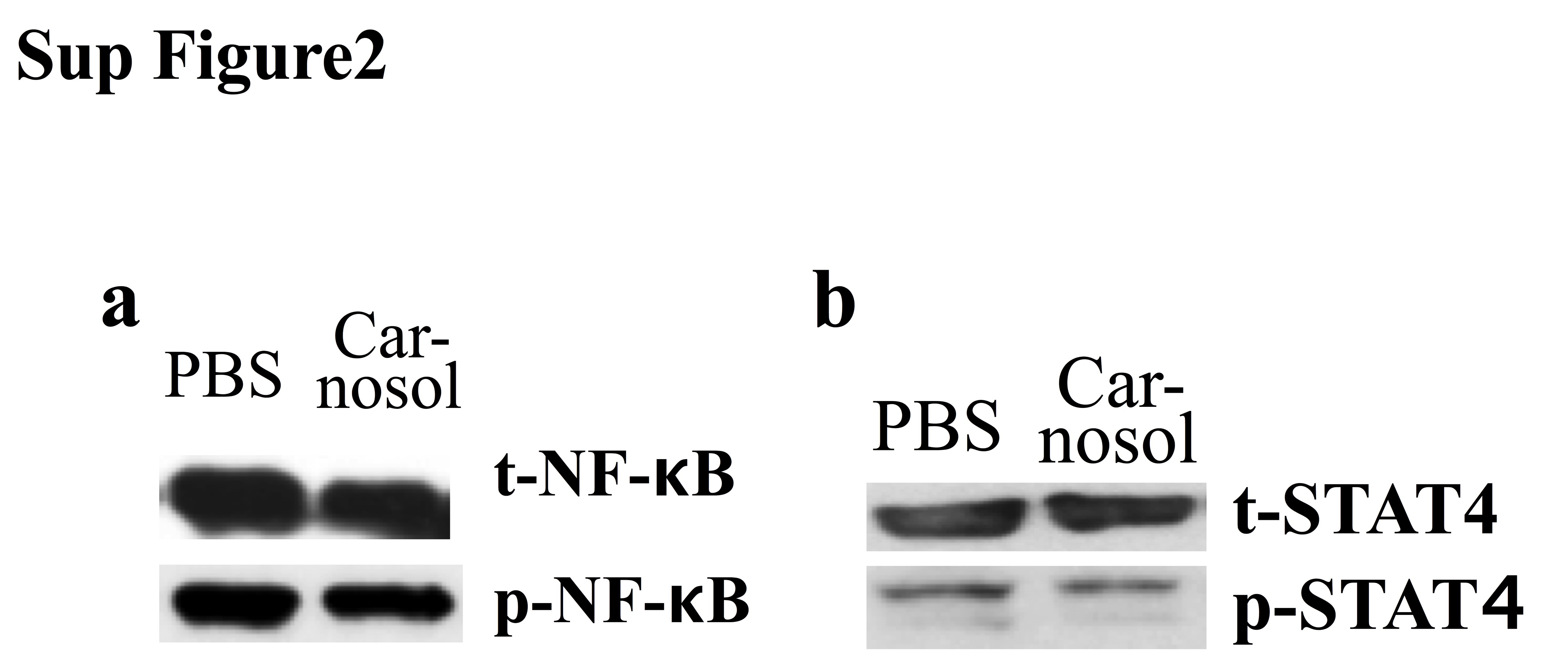


**Sup. Fig. 2. Carnosol treatment did not alter Th1 cell differentiation and NF-κB and STAT3 expression.** CD4+ T cells were cultured under Th1 polarizing condition with soluble anti-CD3e (1 μg/ml), anti-CD28 (1 μg/ml), anti-IL4 (10 μg/ml) and IL-12 (5 ng/ml) and treated with 10 μM carnosol or PBS for 3 days. Cells were then analyzed for NF-κB and STAT4 expression by Western blot.
