# Supplementary material for: Carnosol Modulates Th17 Cell Differentiation and Microglial Switch in Experimental Autoimmune Encephalomyelitis
Source: Front Immunol. 2018 Aug 13;9:1807. doi: 10.3389/fimmu.2018.01807 (PMC6100297; doi:10.3389/fimmu.2018.01807)
Supplement: Supplementary file 2 [file Table_1.docx]

**Supplemental information**

**Primers used for real-time quantitative RT-PCR analysis**

| Gene | Forward | Rewards | |
| --- | --- | --- | --- |
| IL-1β | CTCTCCACCTCAATGGACAGA | | TGCTTGGGATCCACACTCTC |
| IL-17a | TTTAACTCCCTTGGCGCAAAA | | CTTTCCCTCCGCATTGACAC |
| IL-17f | TGCTACTGTTGATGTTGGGAC | | AATGCCCTGGTTTTGGTTGAA |
| IL6 | AGCCAGAGTCCTTCAGAGAGA | | GCCACTCCTTCTGTGACTCC |
| IL12 | AGTGACATGTGGAATGGCGT | | CAGTTCAATGGGCAGGGTCT |
| IL2 | CCTGAAACTCCCCAGGATGC | | TCAAATCCAGAACATGCCGC |
| TNF-α | GACGTGGAACTGGCAGAAGAG | | GCCACAAGCAGGAATGAGAAG |
| NOS II | GCTTGCCCCTGGAAGTTTCT | | CCTCACATACTGTGGACGGG |
